# From Cancer Patient to Provider: An Autobiographical Case Report

**DOI:** 10.7759/cureus.20764

**Published:** 2021-12-27

**Authors:** Taylor L Barnett

**Affiliations:** 1 Hematology/Oncology, Brooke Army Medical Center, San Antonio, USA

**Keywords:** cancer survival, adolescent young adult populations, male fertility, chemotherapy-related toxicity, health education & awareness, ototoxicity, chemoinduced neuropathy, radical orchiectomy, testicular germ cell tumors

## Abstract

Testicular cancer is considered to be the model for the curable neoplasm, with outcomes improving from nearly universal fatality to nearly universal cure in the matter of two decades, driven largely in part by the accidental discovery and application of platinum chemotherapy. Such a diagnosis and treatment can have significant and long-lasting effects on patients, although with every such experience come learning opportunities. This autobiographical case report describes the author’s experience being diagnosed with testicular cancer, the challenges faced during treatment and survivorship, the lessons learned being a patient, and the way they guided him on his path to his current role as an adolescent and young adult (AYA) oncologist.

## Introduction

A few cancers have had such a remarkable history of evolution as had testicular cancer. What once portended a bleak prognosis has become one of the most curable neoplasms in existence. Even before I was working in medicine, I would hear people say that if one were to be diagnosed with any cancer that testicular cancer would be the one to choose. Little did I know that as I was embarking upon my own career in medicine that statement would become all too real. 

I had an organic chemistry professor who would say that in the end when faced with any disease a doctor will always lose. Disheartened does not come close to what a college lecture hall packed full of aspiring physicians like me, just embarking on their scholastic journeys soon to define the rest of their lives, felt that day. Nevertheless, I already knew the road ahead of me was going to be a challenge, not because I desired to prove my professor wrong but rather I wanted to discover for myself what becoming and being a doctor would mean to me. Little did I know such exposure would begin before dawning even the short white coat when I found myself at the other end of the stethoscope just a few days before my college graduation when I was diagnosed with testicular cancer.

This case report details the time I was diagnosed with testicular cancer, my journey through surgery and chemotherapy, and the impact such an experience has made on my life and career as a young oncologist.

## Case presentation

I was a few days away from my university graduation when I found myself at the local emergency room for an evaluation of a daylong history of tinnitus and a frontal headache, along with an occasional gnawing pain in my right testicle for months with a small lump I had noticed about one month prior to presentation. The doctor who examined me noticed a bean-sized fixed yet non-tender hard mass just on the lateral portion of my right testicle. A testicular ultrasound was performed at that time and revealed a heterogenous right testicular mass that was 2cm in diameter. Additional labs were obtained which revealed leukocytosis (white blood cell count of 12.7 x10^3^/mcL) and thrombocytosis (platelet count of 527 x10^3^/mcL), although the rest of the complete blood count and chemistry panel were unremarkable. Unfortunately, I am lacking the information regarding the tumor markers done preoperatively, but according to another physician's report, they were abnormal. This eventually led to computerized tomography (CT) scan of the head, chest, abdomen, and pelvis which showed some scattered enlarged retroperitoneal lymph nodes up to 2cm in diameter, but the rest of the scan, including the head, was unremarkable. I was evaluated promptly by a urologist who recommended a unilateral orchiectomy. Just five hours after checking into the emergency room, I was on an operating room table with a large group of gowned figures surrounding me and a face mask coming toward me as the nurse anesthetist told me to count down from ten. 

I left the hospital the following day and walked across the graduation stage (my then-roommate would rather say I hobbled with a cane) to accept my diploma the next day. However, much to the dissatisfaction of my parents and doctors, I decided to take a month-long trip to Europe with my college friends to celebrate graduation before doing any further therapy. 

By the time I had a follow-up appointment with an oncologist almost seven weeks had passed since my orchiectomy. Repeat CT scans, unfortunately, showed new metastases above the diaphragm in some thoracic nodal areas, most notably a mediastinal node measuring 2.3cm. There was also a 1.5cm lung nodule concerning malignancy, although this was never biopsied. There was the involvement of neither the spermatic cord nor the scrotal wall. Final pathology from the right orchiectomy revealed a mixed germ cell tumor limited to the testis replacing the upper two-thirds of the right testicle. Also identified were a large embryonal component (approximately 95%) and the presence of lymphovascular invasion (both were the features that can indicate a higher risk of relapse). Post-orchiectomy tumor markers remained elevated, with lactate dehydrogenase of 235 U/L, alpha fetoprotein (AFP) of 952 ng/mL, and beta-human chorionic gonadotropin (bHCG) of 1457 mIU/mL. 

These aforementioned changes staged me at Good-Risk Stage IIIA disease (pT2/N2/M1a/S1 per most current American Joint Committee on Cancer Eighth Edition staging criteria). That classification of good risk did little to soothe me when I learned that with testicular cancer there was no such thing as Stage IV disease. I promptly signed consent for chemotherapy and started three three-week cycles of adjuvant bleomycin/etoposide/cisplatin (BEP) chemotherapy on my birthday. I refused an implanted port-a-cath and instead elected to have all agents administered through peripheral intravenous catheterization, a decision which I later regretted due to the discomfort and subsequent difficulty of access with vessel irritation. 

Chemotherapy did come with complications. I had an episode of neutropenic fever with meningitis during my second cycle but recovered. There was discussion of granulocyte colony-stimulating factor (G-CSF) support during my last cycle but such was not ultimately pursued. 

After finishing three cycles of adjuvant chemotherapy, subsequent tumor markers showed complete normalization and a CT of the chest, abdomen, and pelvis showed no residual disease. Based on my experiences, I chose a career in medicine and am now an AYA oncologist. Now over thirteen years out from my diagnosis, I am glad to say I remain in remission.

## Discussion

As I encourage my fellows and colleagues, we should have a duty to learn from our experiences in order to benefit ourselves and others. All of the events that happened to me during treatment, including those of my own mistakes, have taught me not only about cancer itself but also about what I could do in my life with such knowledge and experiences, leading me eventually to where I am today. 

With regard to my delay in diagnosis, I know I am to blame but I can understand what patients go through during such a trying time, and I encourage my patients not to delay evaluation for any concerning abnormality and uncertainty. As I received, the radical inguinal orchiectomy with high ligation of the spermatic cord at the level of the internal ring is indeed the procedure of choice for surgical management of testicular cancer as it establishes the histopathologic tumor type while providing definitive local tumor control with minimal morbidity. Other attempts such as partial orchiectomy sometimes done to preserve fertility but with the risk of leaving behind microinvasive disease or trans-scrotal approaches, which can distort lymphatic drainage with a perhaps increased risk of recurrence, have been done in select cases with optimal management especially with regards to adjuvant therapy have not been defined [1]. As such, these procedures are not often performed. While there is no standard time interval between orchiectomy and adjuvant therapy, on average this seems to be less than one month in my experience. Waiting can be important in some cases, for the post-surgery tumor marker levels are crucial for the serologic “S” staging of testicular cancer that can impact prognosis and treatment. As an oncologist, I usually check weekly levels, for bHCG has a half-life of 24 to 48 hours while that of AFP is five to seven days [2]. Rapid normalization of serum bHCG within two weeks and AFP within 25 to 30 days suggests the elimination of the tumor, while the persistent elevation of tumor markers after the period during which normalization is expected to occur may suggest persistent occult disease even in spite of negative imaging.

The side effects of cisplatin are fairly notorious. I was fortunate to have not much nausea or vomiting because of my aggressive antiemetic prophylaxis regimen with aprepitant, dexamethasone, and ondansetron. Beyond antiemetic drug development, the renal toxicities have over the years become more manageable. Electrolyte imbalances, most prominently with potassium and magnesium, arise from cisplatin-induced renal tubular toxicity. Aggressive hydration is key, and while there are numerous recommendations of the use of electrolyte supplementation, I usually follow the guidance of the British Columbia Cancer Agency Genitourinary Tumour Systemic Policy Group shown in Table 1 with some modifications I typically made. I note that if patients have had chronic electrolyte issues I usually give them standing oral replenishment as well as part of their outpatient medications. I avoid co-administration of mannitol as a randomized control study showed it did not prevent acute nephrotoxicity in patients receiving cisplatin [4]. One must take into account other comorbidities and overall volume status when determining the hydration plan, as such, each case must be individualized. 

**Table 1:** Hydration plan for cisplatin Cisplatin hydration and electrolyte additives are shown in the table. Note that volume may include hydration associated with the administration of other drugs (e.g., other chemotherapy agents, supportive intravenous [IV] medications) [3]

Cisplatin (mg/m^2^)	Hydration	Electrolyte additives	Comments
Greater than 80	3 liters	Potassium Chloride 20-40mEq + Magnesium Sulfate 2g	-
Between 60-80	2 liters	Potassium Chloride 20mEq + Magnesium Sulfate 2g	-
Between 40-60	1 liter	Potassium Chloride 20mEq + Magnesium Sulfate 1-2g	includes regimens with cisplatin administered over multiple days
Less than 40	0.5 liter	None (or oral supplementation as needed)	includes regimens with cisplatin administered over multiple days

Cisplatin also causes significant neurotoxicity, possibly due to the apoptotic damage to nuclear and mitochondrial DNA in the dorsal root ganglion [5]. Even if cisplatin is discontinued, the sensory neuropathy may continue to worsen for several months in 30% of patients. Most patients recover eventually, although in a study of long-term testicular cancer survivors who had completed treatment at least five years previously, peripheral neuropathy was still present in 20% of patients, and 10% were symptomatic [6]. There are unfortunately no effective preventive methods to reduce one's risk of neuropathy. I get Raynaud’s phenomenon and have peripheral neuropathy in my toes which is noticeable but not bothersome. Ototoxicity is characterized by a dose-dependent, high-frequency sensorineural hearing loss, which is almost always bilateral and irreversible, and often accompanied by tinnitus and vertigo. Hearing loss has been found in as many as 80% of testicular cancer survivors, with up to 18% with severe or profound hearing loss [7]. Many advocate for frequent monitoring and early detection, and the use of sodium thiosulfate has been shown to be safe and efficacious in children receiving cisplatin monotherapy for standard-risk hepatoblastoma [8]. Nevertheless, evidence is insufficient to support the routine use of such in children receiving treatment of other malignancies, adults receiving cisplatin, or for any other agent (such as amifostine and vitamin E) to prevent cisplatin ototoxicity. I have occasional episodes of tinnitus and unfortunately have had a decline in my hearing with high-pitched sounds, but I am lucky not to need hearing aids and I continue to practice preventive measures as I recommend to patients such as avoiding loud environments and wearing earplugs. 

Bleomycin is well known for its potential for life-threatening interstitial pulmonary fibrosis (also called fibrosing alveolitis) in up to ten percent of patients, with a lesser extent of patients experiencing other pathologic processes such as organizing pneumonia and hypersensitivity pneumonitis [9]. I experience still a chronic hacking cough with some mild yet noticeable radiographic pulmonary scarring, but I was lucky not to have any advanced disease during treatment. Bleomycin hydrolase, an enzyme that degrades bleomycin, is active in all tissues with the exception of the lung (as well as the skin), which may account for the specific pulmonary toxicity as the drug interacts with alveolar macrophages to release inflammatory cytokines and oxygen radicals [9]. While early data suggested a high rate of fatality associated with bleomycin-induced pulmonary injury (BPI), more recent data suggest that the overall risk of clinically apparent and fatal BPI may be lower, and that BPI is reversible and without long-term sequelae in most cases. In a Danish study of 565 patients (50% with a smoking history, considered one of the major risk factors for BPI) with germ cell tumor treated with BEP, only 9% discontinued bleomycin because of a significant (greater than or equal to 25%) decline in hemoglobin-corrected diffusing capacity for carbon monoxide (DLCO). At 16.1-year follow-up, in comparison with a contemporaneously treated group of 2548 patients with stage I germ cell tumors who received no chemotherapy and instead underwent surveillance, the 15-year cumulative risk of pulmonary fibrosis (0.4% in the BEP group vs. 0.2% in the non-BEP group) and obstructive pulmonary disease (3.3% vs. 2.8%) were overall quite low and similar [10]. While pulmonary function normalized in the majority over time, patients who underwent pulmonary surgery had continuously decreased pulmonary function as did smokers. There were no differences in long-term outcome among patients who received all of the doses of bleomycin (median dose 142 IU/m^2^) versus those whose doses were attenuated because of changes in the DLCO (median dose 100 IU/m^2^) [10]. As such, in patients who have asymptomatic decreases in DLCO, early discontinuation of bleomycin leads to low rates of long-term bleomycin-induced pulmonary disease without compromising oncologic outcomes. Smoking cessation also is critical, as is peri-operative management with minimization of supplemental oxygen and avoidance of excess intravenous fluid, and I notified my proceduralist of my history before I receive supplemental oxygen or anesthesia. As was considered for me, G-CSF has also been identified as a possible risk factor for the development of bleomycin-induced pulmonary injury (BPI) in various animal studies, and one retrospective analysis, in particular, showed BPI to have a negative impact on survival in patients with Hodgkin Lymphoma [11]. However, such has not been proven in other studies including a randomized trial showing that G-CSF is safe and effective at maintaining dose intensity and timing of BEP without any significant increase in BPI or negative impact on survival [12].

Survivorship is a critical issue for patients treated for testicular cancer. With more patients living after their treatment, appropriate surveillance and management of side effects and long-term health risks associated with treatment are vital. Fortunately, I am over 13 years out from treatment with no evidence of recurrence. I actually do not see an oncologist anymore but I do, as I advise my patients, to continue to perform regular testicular exams and improve one’s overall health even after their formal surveillance ends, with regular visits with their primary care doctor. The crucial aspect of survivorship health maintenance was demonstrated with recent data of a study of 5707 men treated for testicular cancer showing that based on standard mortality ratios there is an overall excess non-testicular cancer-related mortality of 23% compared with the general population, especially in those patients who received radiation and/or at least two cycles of platinum-based chemotherapy, although not with those who underwent surgery alone [13]. The highest mortality was seen in those who were less than 20 years of age at diagnosis (with a 2.27-fold significantly increased risk compared to a normal population) and increasing beyond ten years of follow-up time, with the most common cause of death due to secondary malignancy with a 53% excess mortality. Overall, non-cancer mortality was increased by 15%, with the risk of suicide significantly increased after platinum chemotherapy compared to the general population. Cardiovascular health is also very important for survivors, especially for me with my personal history of familial hypercholesterolemia and my family history of extensive cardiovascular disease and sudden cardiac death. In a study of 2512 testicular cancer survivors, the overall standardized incidence ratio for myocardial infarction and angina pectoris was 1.17 (95% confidence interval of 1.04 to 1.31) in men younger than 45 years compared to healthy controls [14]. Testicular cancer survivors also have an almost two-fold higher risk of metabolic syndrome compared with controls [15]. In addition to having taken statins since my teens, I use a nutrition tracker to maintain a balanced diet and do high-intensity interval training up to six times a week to keep a healthy weight. Chemotherapy has made me infertile but I am not seeking to have children. Nevertheless, I encourage my young patients even with no immediate desire to have children to consider fertility preservation seriously as sometimes after treatment it may be too late, which can pose significant psychosocial issues later in life.

Indeed, some of the most meaningful events can occur by accident, and the history of the treatment of testicular cancer is by no means different. Dr. Barnett Rosenberg was a chemistry and biophysics professor at Michigan State University who in the 1960s had noticed that images of dividing cells resembled the pattern of iron shavings subjected to a magnetic field. He wondered whether this phenomenon meant that an electrical field could also affect cell division, so he devised an experiment to test his curiosity [16]. As platinum was thought to have no biological activity, Dr. Rosenberg and his colleagues placed platinum electrodes into a solution containing *E. coli* to observe its growth. When an electrical current was applied the bacterial cell division halted, which resumed when the current was stopped. While it was thought that the electrical field was controlling cell division, it turned out that certain group VIIIb transition metal compounds (namely [(NH_4_)_2_PtCl_6_]) were released from the electrodes and underwent photochemical changes in the bacterial medium to produce cis-[Pt(NH_3_)_2_Cl_2_]. This chemical was thought to be the key factor, which according to Dr. Rosenberg himself, led to the "accidental discovery that led eventually to cisplatin." After he published these initial findings to be followed up four years later with findings showing cisplatin could cure murine sarcoma 180 and leukemia L1210 in mouse models [17]. 

About a decade after this research, Dr. John Donohue, a urologist at Indiana University Medical School, welcomed to the faculty 32-year-old Dr. Lawrence Einhorn, who had devoted his career to improving outcomes for those with testicular cancer. Previously, the retroperitoneal lymph node dissection for early-stage testicular cancer was the modality of choice to achieve the best outcomes, with a 20% overall survival rate [18]. However, if metastatic, testicular cancer resulted in a uniformly fatal prognosis with an abysmal overall survival rate of 5% [18]. Going based on data from prior phase one studies of cisplatin, Dr. Einhorn combined a dose of cisplatin 20mg/m^2^ daily for five days with bleomycin and etoposide providing a cure rate of 83% in de novo disease and 25% in refractory disease [19], representing a marked improvement in outcomes in front-line therapy and the first time an adult solid tumor was cured with second-line chemotherapy. Nearly just one decade after Rosenberg’s original publication, in 1978 cisplatin was approved by the US Food and Drug Administration for clinical treatment of genitourinary tumors and has helped make what was once a nearly universally fatal disease now curable in up to 98% of patients with over 80% enjoying long-term survival [20]. 

## Conclusions

Looking back at the time when I was diagnosed, I was truly ignorant of the gravity of the disease I had. Although as I went along my journey with cancer I tried to learn all I could about it, it did not become solidified nor reach the highest level of meaningfulness until I saw my first patient with testicular cancer early in my first year of fellowship. All of the residual treatment-related health issues still present today, the times of doubt and uncertainty as I entered the CT scan for each surveillance scan, the innumerable late nights I passed in solitude in my medical school’s library while studying for the next of a multitude of exams, the staggering tuition I paid to invest in my education and achieve my medical degree, and the rejoice of the highs and the heartbreak of the lows -- all of that was worth it as things seemed to have come full circle to this one moment when I sat across from this man, the same age I was when I was diagnosed, to follow him along in his own journey into battle just as I had done eight years prior. 

Overall, the burden of treatment may linger, but it all has been the most impactful experience on my life. As I tell my patients, I have learned that cancer (unlike what I did) takes no holidays and at the end of the day will do what it wants, and with it and treatment there are no guarantees. I have developed a deep understanding and empathy for my patients who go through their own struggles with cancer and chemotherapy. I am inspired to learn more about their disease processes, stay current with the latest developments, and teach my fellows what I have learned and experienced as they become colleagues of mine in what I consider to be the most challenging yet rewarding field of medicine. We should see everything that may happen to our patients, loved ones, and even ourselves as an opportunity to better ourselves and our understanding of how things work and evolve, both in medicine and in life. A better understanding of such will become important when we may become the ones lying in the hospital bed instead of standing at the foot of it.

While some may see a previous cancer diagnosis as a burden, I see it as a badge of honor (quite literally with the orchid-colored ribbon I have on my hospital badge) and to this day I continue to share my story with my patients. Owning one’s challenges builds strength and character, and I encourage the readers with any similar experiences to share them with their patients. It can give them at their most trying times what they really need, more than any chemotherapy, surgery, or radiation. It gives them a human connection. With that, no one loses. 
